# Woods and Russell, Hill, and the emergence of medical statistics

**DOI:** 10.1002/sim.3893

**Published:** 2010-05-14

**Authors:** Vern Farewell, Tony Johnson

**Affiliations:** a Medical Research Council Biostatistics Unit, University of Cambridge Institute of Public Health, University Forvie SiteRobinson Way, Cambridge CB2 0SR, U.K.; a Medical Research Council Clinical Trials UnitLondon, U.K.

**Keywords:** medical statistics, Austin Bradford Hill, Hilda Mary Woods, William Thomas Russell

## Abstract

In 1937, Austin Bradford Hill wrote *Principles of Medical Statistics* (Lancet: London, 1937) that became renowned throughout the world and is widely associated with the birth of modern medical statistics. Some 6 years earlier Hilda Mary Woods and William Thomas Russell, colleagues of Hill at the London School of Hygiene and Tropical Medicine, wrote a similar book *An Introduction to Medical Statistics* (PS King and Son: London, 1931) that is little known today. We trace the origins of these two books from the foundations of early demography and vital statistics, and make a detailed examination of some of their chapters. It is clear that these texts mark a watershed in the history of medical statistics that demarcates the vital statistics of the nineteenth and early twentieth centuries from the modern discipline. Moreover, we consider that the book by Woods and Russell is of some importance in the development of medical statistics and we describe and acknowledge their place in the history of this discipline. Copyright © 2010 John Wiley & Sons, Ltd.

## 1. Introduction

Between 2 January and 24 April, 1937 *The Lancet* published a series of 17 articles on medical statistics by Austin Bradford Hill; the series was suggested by Dr M H (Pamela) Kettle, an assistant editor at *The Lancet*, and Hill was paid £3.3.0 [1] (three pounds, three shillings, and no pence, and roughly equivalent to £135 today) for each ‘instalment.’ The 17 articles, with an additional set of definitions and a reproduced table of χ^2^, formed the chapters of *Principles of Medical Statistics* [2], published very soon thereafter on 17 June 1937, which over the next 54years was to run through 12 editions, and become renowned worldwide among physicians, epidemiologists, and medical statisticians. A list of the articles is given in our supplementary material.‡

Hill joined the department at the London School of Hygiene and Tropical Medicine (LSHTM) headed by Major Greenwood in 1927 after obtaining an honours degree in economics, and then a grant from the Medical Research Council to examine the high mortality in young adults in rural areas of England. While carrying out this study, he attended Karl Pearson's course on statistics at London University. By the end of 1936 Hill had published 39 book reviews, 8 research reports, and 16 papers (including nine in the *British Medical Journal* or *Lancet*, and four in the *Journal of the Royal Statistical Society*). Many of the reviews were on books about population, poverty, industrial working conditions, migration, mortality, and the social conditions in London, subjects of obvious central interest to LSHTM. However, he also reviewed Hartshorne and May's *Studies in Deceit*, Burgess's *Introduction to the Mathematics of Statistics*, Fisher's *The Genetical Theory of Natural Selection*, and the second edition of Pearl's *Introduction to Medical Biometry and Statistics*, thus demonstrating an interest extending into statistics, genetics, and beyond. Most of his papers also reflected a focus on mortality, longevity, social conditions, industrial working conditions, including nursing, thereby in part reflecting the vital statistics of the previous 100 years, but extended beyond these to cricket, experimental epidemiology, and the openings into medical practice.

Where did Hill develop the ideas that were to lay the foundations of his famous book? Did they develop from his own research studies, his teaching, or perhaps through discussions with, or publications of, his colleagues? The first, his own research studies, is established in the Preface to the first edition [2] where Hill remarks ‘The worker in medical problems, in the field of clinical as well as preventive medicine, must *himself* know something of the statistical technique, both in experimental arrangements and in the interpretation of figures. To enable him to acquire some knowledge of this technique I have tried to set down as simply as possible the statistical methods that experience has shown me to be most helpful in the problems with which medical workers are concerned. I have used examples taken from medical inquiries in the attempt to make clear these methods of analysis, and have sought to show by illustration where and why workers make mistakes in their interpretation of figures. With respect to the second, his teaching, in the decade starting in 1927 Hill would have taught statistics to medical students at LSHTM though exactly what these courses covered we cannot be sure. He does not refer to them in the preface to his book although Sir Richard Doll did link them to the book in his *Encyclopedia of Biostatistics* [3] entry on Hill. Undoubtedly, however, Hill must have drawn somewhat on his teaching experience. Peter Armitage (personal communication) recalls that in the late 1940s Hill lectured to Diploma students, mostly medical, with the content being based on Hill's book and had the impression that similar lectures had gone on before World War II.

In this paper we focus particularly on the third, his collegial links within LSHTM, by considering a comparatively little known text by Hilda Mary Woods and William Thomas Russell, both colleagues of Hill. As a prelude we attempt to trace the origins of the early textbooks on statistics, and on vital and medical statistics more specifically, from the seventeenth to the early twentieth centuries. This will provide a sense of the body of material to which Woods and Russell's *An Introduction to Medical Statistics* [4] and Hill's *Principles of Medical Statistics* [2] added. Further, we make some detailed comparisons between these two books and provide information on their histories of publication. Note that our focus is primarily on textbooks and it is beyond the scope of this paper to deal in detail with the variety of other writings relevant to the development of medical statistics. For example, Dunn [5] published an almost book length review of statistical methods motivated by the lack of use of such methods in the physiological literature.

## 2. Early history and textbooks

The roots of medical statistics were apparent in the 1930s and, as suggested by Colton *et al*. [6], encompass the straddling of two quite different branches of knowledge with the deployment of the ideas, principles, and methods of statistics to stimulate a deeper understanding of medicine. Here, we provide a brief overview of this development in the U.K. and have borrowed freely from several sources including Armitage [7], Chen [8], Gluud [http://www.jameslindlibrary.org/trial_records/19th_Century/heiberg/heiberg-commentary.html], Greenwood [9, 10], Magnello and Hardy [11], Nissel [12], and Sheynin [13]. Pearson [14] also provides an interesting insight into this early history.

One starting point was the accumulation of regular, brief records by an injunction of Thomas Cromwell in 1538 that required the clergy of every parish to keep registers of church baptisms, weddings, and funerals. Ultimately however, as Nissel [12] states, ‘producing national statistics was no business of the Church, nor could it easily be fitted into its administrative framework’, and the system floundered until the establishment of a civil registration system in 1837.

A second starting point was the London Bills of Mortality collected sporadically from the early sixteenth century, and weekly from 1603 to 1836; they were collected by parish clerks and published weekly. Their original purpose was to provide an early warning of plague to inform public health measures (such as those mentioned by Defoe [15]). Initially the Bills reported separately the numbers of deaths from plague and from all other causes combined, but were extended by 1570 to include baptisms, from 1629 to give cause of death, and from the early eighteenth century, the age at death.

These two sources of information were the foundation of the political arithmetic of William Petty (1623–1687) and the early demography of John Graunt (1620–1674).

The third starting point was the census of population introduced in England, Scotland and Wales in 1801, as a result of the pioneering work of John Rickman (1771-1840), who drafted the Census Act of 1800, and conducted the censuses up to 1831. As Nissel [12] states, census taking has a long history that extends back to ancient civilizations including the Babylonians, Egyptians, and Chinese, and was undertaken by several European countries from the early 1700s though not necessarily on a regular basis. However, Godfrey [16] credits Canada with ‘the first nominal census of modern times, that is to say, a record for each individual by name’, undertaken in 1666. Knibbs[17] provides a valuable early history of censuses and their details.

By 1850 accumulation of data about individual cities, communities, regions, and countries, around the world provided a considerable quantity of information that was available for detailed study and analysis including comparisons between different locations, and changes with time. This mass of data was eventually subsumed under the title *vital statistics* (attested from 1837), that encompassed the important events of life, specifically births, marriages, and deaths, and more generally, health and disease, migration, and economic activity. These data sources enabled the work of William Farr (1807–1883) in the General Register Office, whose thinking was influenced by the French writers Pinel (1745–1826), Louis (1787–1872), and Gavarret (1809–1890); see Armitage [7].

### 2.1. Vital statistics

Table I provides a timeline for the publication of 66 textbooks on vital and medical statistics up to 1937, the year that Hill's papers appeared in *The Lancet;* we describe some of these briefly. (We have restricted our attention to books since we consider that these provide a wider and more permanent indication of the historical development of this subject than published papers; we have excluded textbooks on probability applied to medicine).

It is difficult to be precise about the first textbook on vital statistics since these started to appear from the start of the nineteenth century (and perhaps before) in several countries and languages, and consequently we do not claim that Table I provides a complete list. Two textbooks on vital statistics which also included ‘medical statistics’ in their titles appeared within 4years of one another, in 1825 and 1829. Johann Ludwig Casper (1796–1864), who became professor of forensic medicine in Berlin, published his work in three parts (1825, 1835, and 1846); the first covers suicide, the poor and sick in Paris, and mortality of children in Berlin [18]. For this volume he was awarded a diamond ring by the King of Prussia, and a reviewer described it as ‘one of the most interesting contributions to medical statistics that has appeared during the present century; there is not much novelty in it, but it establishes upon satisfactory evidence many points that have been merely matters of doubtful opinion, and it settles some important questions regarding which there has been a keen controversy among physicians and political economists’. The second volume (1835) is remarkable for a paper on the probable duration of life, whereas the third (1846) includes communications on the influence of the weather upon the health and mortality of mankind, upon the geography of crime, on the influence of the period of the day upon the birth and death of man, and an essay upon pyromania. Further details can be found in the biographical notice by Balfour [19].

**Table I:** Timeline of textbooks on vital and medical statistics to 1937.

Year	Author	Title and edition
1805	Donnant	*Théorie elementaire de la statistique*
1805	Peuchet	*Statistique élémentaire de la France*
1824	Mone	*Theorie der Statistik*
1825	Casper	*Beiträge zur medicinischen Statistik und Staatsarzneikunde. (vol. I)*
1829	Hawkins	*Elements of Medical Statistics*
1834	Mone	*Théorie de la Statistique*
1835	Casper	*Beiträge zur medicinischen Statistik und Staatsarzneikunde. (vol. II)*
1840	Dufau	*Traité de Statistique (abbrev.)*
1840	Gavarret	*Principes Généraux de Statistique Médicale (abbrev.)*
1845	Neison	First edition *Contributions to Vital Statistics (abbrev.)*
1846	Casper	*Beiträge zur medicinischen Statistik und Staatsarzneikunde. (vol. III)*
1846	Neison	Second edition *Contributions to Vital Statistics (abbrev.)*
1847	Heuschling	*Manuel de statistique ethnographique universelle*
1855	Guillard	*Eléments de statistique ou démographie comparée*
1856	Laycock	First edition *Principles and Methods of Medical Observations (abbrev)*.
1856	Moreau de Jonnes	*Eléments de Statistique*
1857	Boudin	*Traité de géographie et de statistique médicales et des maladies endémiques (abbrev.)*
1857	Neison	Third edition *Contributions to Vital Statistics (abbrev.)*
1859	Bryson	*Medicine and Medical Statistics*
1860	Block	First edition *Statistique de la France comparée avec les autres états de l'Europe*
1860	Boudin	*Éléments de statistique et de géographie générales*
1864	Laycock	Second edition *Principles and Methods of Medical Observations (abbrev.)*
1865	Oesterlen	*Handbuch der Medicinischen Statistik*
1869	Orlandini	*Elementi di statistica*
1874	Hirschberg	*Die Mathematischen Grundlagen der Medicinischen Statistik*
1874	Peskov	*Medical Statistics and Geography (in Russian)*
1875	Block	Second edition.*Statistique de la France comparée avec les autres états de l'Europe*
1878	Block	First edition *Traité Théorique et Practique de Statistique*
1880	Block	Second edition *Traité Théorique et Practique de Statistique*
1881	Westergaard	*Die Lehre von der Mortalitat und Morbilität Anthropologisch-Statistiche Untersuchungen*
1885	Farr	*Vital Statistics*
1886	Block	Second edition (revised) *Traité Théorique et Practique de Statistique*
1889	Calatabiano	*La statistica teorica e applicata*
1889	Newsholme	First and second edition *The Elements of Vital Statistics*
1889	Sorensen	*Ledetraad for Laeger ved statistiske Undersogelser*
1892	Newsholme	Third edition *The Elements of Vital Statistics*
1895	Bertillon	*Cours Elementaire de Statistique Administrative*
1899	Newsholme	Third edition (rewritten). *The Elements of Vital Statistics*
1901	Benini	*Principii di demografia*
1904	Colajanni	*Manuale di statistica teorica e demografica*
1904	Macleod	*Methods and Calculations in Hygiene and Vital Statistics*
1906	Broggi	*Matematica Attuariale*
1906	Prinzing	*Handbuch der Medicinischen Statistik*
1909	Aldo	*Teoria statistica generali e demografia*
1914	Burn	*Vital Statistics Explained: Some Practical Suggestions*
1917	Knibbs	*The Mathematical Theory of Population*
1919	Whipple	First edition *Vital Statistics: An Introduction to the Science of Demography*
1923	Falk	*The Principles of Vital statistics*
1923	Feldman	First edition *Biomathematics*
1923	Newsholme	Third (new edition). *The Elements of Vital Statistics* (abbrev.)
1923	Pearl	First edition *Introduction to Medical Biometry and Statistics*
1923	Whipple	Second edition *Vital Statistics: An Introduction to the Science of Demography*
1924	Newsholme	Fourth edition *The Elements of Vital Statistics*
1930	Freudenberg	*Die Statistischen Methoden* (Biological statistics)
1930	Pearl	Second edition *Introduction to Medical Biometry and Statistics*
1931	Prinzing	Second edition *Handbuch der Medicinischen Statistik*
1931	Tippett	First edition *The Methods of Statistics* (Biological Statistics)
1931	*Woods and Russell*	*First edition An Introduction to Medical Statistics*
1933	Davies and Crowder	*Methods of Statistical Analysis in the Social Sciences*
1934	Snedecor	First edition *Calculation and Interpretation of Analysis of Variance and Covariance*
1935	Davis and Nelson	First edition *Elements of Statistics with Application to Economic Data*
1935	Feldman	Second edition *Biomathematics*
1936	*Woods and Russell*	Second *edition An Introduction to Medical Statistics*
1937	Davis and Nelson	Second edition *Elements of Statistics with Application to Economic Data*
1937	Tippett	Second edition *The Methods of Statistics* (Biological Statistics)
1937	*Hill*	*Lancet papers and First edition Principles of Medical Statistics* (abbrev. indicates abbreviated title)

The volume by Francis Bisset Hawkins, MD (1796–1894) was published in 1829 [20]. His varied career and achievements have been acknowledged by the Royal College of Physicians in London who in 1899 founded the Bisset Hawkins Medal awarded triennially for advances in sanitary science and public health.

The book is basically a summary of a collection of diverse reports, the author suggesting in his preface that ‘a favourable moment has, perhaps, at length arrived for arranging these scattered fragments into the rudiments of a system, and for comparing together, in close apposition, the documents afforded by different countries and institutions, which at present lie far asunder’. Greenwood ([10], p. 69) was unimpressed suggesting that Hawkins' work was ‘tendentious’ and ‘uncritical,’ although the author deserves credit for two notable achievements. One was providing the first definition of medical statistics—*the application of numbers to illustrate the natural history of man in health and disease*—which along with the content of the book reflects what is more properly labelled as vital statistics. The other was his vision of the application of statistics ‘in reference to the natural history of man in health and disease [that] would materially assist the completion of a philosophy of medicine, by pointing out to the physicians of every part of the world the comparative merits of various modes of practice, the history of diseases in different ages and countries, the increase and decrease of particular maladies, the tendency of certain situations, professions, and modes of life to protect or to expose; and by indicating, as the basis of prognosis, those extended tabular views of the duration and termination of diseases, which are furnished, at successive periods, by hospitals and civic registers’; thus presaging the enormous progress that would ensue from the setting-up of the registration of births, marriages, and deaths a decade later in U.K., and elsewhere. The book is chauvinistic as indicated by the last chapter that sets out Hawkins' conclusions that, in England, men, animals, and vegetables all flourished because of the care bestowed on their culture, resulting in a country that was the most healthy with which he was acquainted!

Medical statistics is also included in the title of Alexander Bryson's writing, which comprised a chapter of 19 pages in the longer work compiled by John Frederick William Herschel (1792–1871) entitled *A Manual of Scientific Enquiry prepared for the use of Her Majesty's Navy: and adapted for travellers in general* [21]. This work appeared in five editions from 1849, with a facsimile of the second edition printed in 1974. The manual was intended to conduce an interest in science among officers, and more particularly medical officers, of the navy, ‘when upon foreign service’. Its contents span Astronomy, Hydrography, Magnetism, Meteorology, Ethnology, Geology, Seismology, Zoology, Botany, Statistics, and Medical statistics. The chapter on statistics (by GR Porter, later corrected by W Newmarch) is a basic argument for the collection of vital, social, and economic statistics, whereas that on medicine and medical statistics (by Bryson, later revised by William Aitken) largely covers the environment (temperature, light, air pressure, and movement) with particular reference to conditions on board ship, and diseases, especially those likely to be encountered by the crew. There are no tables, and indeed, no notable figures in either chapter, but then Herschel (perhaps rather the Lords Commissioners of the Admiralty) was intending to reach a wide audience.

Maurice Block (1816–1901) published, in French, two relevant books, the second [22] dealing with general theory. Two books in German [23, 24], despite their titles including medical statistics, are also books on vital statistics.

At the turn of the century, Herbert WG Macleod produced a short textbook [25], of 144 pages, that according to its preface explains ‘the most important calculations and formulae connected with hygiene and vital statistics’ as a ready reference for ‘medical men and others working at public health'. It is clearly not a work on medical statistics *per se* however.

A number of books with the term ‘Vital Statistics’ in their title subsequently emerged. For example, Burn [26] provides a useful guide to the census and its derived statistics but does not expound outside this narrow area.

A book [27] that was recommended for further reading by Woods and Russell, was by George Chandler Whipple (1866–1924), Professor of Sanitary Engineering at Harvard. The content is similar to the earlier text by Burn (demography, census, birth, death, and marriage rates, standardization, death by cause, life tables) but also includes chapters on probability and correlation, and, with over 500 pages, is three times the length; it was intended for students training to become public health officials, and based principally on the census taken in U.S.A. in 1910.

The second book recommended for further reading by Woods and Russell is by Sir Arthur Newsholme (1857–1943), the first edition of which appeared in 1889 [28]. A second edition was published in the same year with a third edition (almost entirely rewritten) in 1899; this in turn was rewritten and republished in 1923. Newsholme was a fellow of the Royal Statistical Society, medical officer of health in Clapham, then Brighton, the Principal Medical Officer of the Local Government Board in London, lecturer at the School of Hygiene and Public Health at Johns Hopkins University, U.S.A., and also held other distinguished appointments. The 1923 edition covers the vital statistics of both the U.K. and U.S.A., and at over 620 pages it is the most comprehensive of any of the texts on vital statistics. It was written for the use of medical officers of health and others engaged in public health and social work, ‘who have to handle statistical data’. The first part covers familiar topics in vital statistics but the second part is methodological covering elementary statistics, causation, correlation, graphic representation of data, and statistical fallacies.

Falk, who was in the Department of Public Health at Yale in the U.S.A., wrote his 1923 book [29] based on teaching students in the public health nursing course; it avoids theory and methods and is ‘almost wholly devoted to the results of vital statistics.’ Falk acknowledges the texts by both Newsholme and Whipple, and, in common with the first, includes a chapter on the interpretation of statistics including statistical errors and fallacies.

There is a plethora of other texts related to vital statistics published up to 1937 demonstrating clearly that the pioneering work of Graunt (1620–1674), Farr (1807–1883), and others had borne fruit and that the value of routinely collected data was now apparent. Three centuries of data collection and its reporting provided the basis for the subsequent development of medical statistics.

### 2.2. General statistics

Table II provides a timeline of more general textbooks on statistics published up to 1937, some of which we mention briefly. An expanded version is given in our supplementary material. We have excluded texts on probability and we do not claim to have traced all general texts. We also draw attention to a recent study of 56 treatises on statistics published between 1800 and 1940 by Armatte [30].

**Table II:** The timeline of textbooks on statistics (first editions) to 1937.

1861	Airy	*On the Algebraical and Numerical Theory of Errors of Observations and the Combination of Observations*
1880	Gaetano	*Elementi di statistica teorica: principi generali*
1886	Meitzen	*Geschichte, Theorie, und Technik der Statistik*
1889	Thiele	*Forelæsninger over Almindelig Iagttagelseslære*
1890	Westergaard	*Grundzüge der Theorie der Statistik*
1895	Mayr	*Theoretische Statistik*
1897	Thiele	*Elementœr Iagttagelseslœre*
1899	Davenport	*Statistical Methods with Special Reference to Biological Variations*
1901	Bowley	*Elements of Statistics*
1903	Thiele	*Theory of Observations*
1905	Liesse	*La Statistique: ses difficultés, ses procédés, ses résultats*
1906	Benini	*Principii de Statistica Metodologica*
1906	Blaschke	*Vorlesungen uber Mathematische Statistik*
1906	Elderton	*Frequency Curves and Correlation*
1906	Faure	*Eléments de Statistique*
1906	Gaetano	*Elementi di statistica metodologica*
1907	Luigi	*Principi fondomentali di statistica*
1909	Elderton	*Primer of Statistics*
1909	Thiele	*Interpolationsrechnung*
1910	Bowley	*An Elementary Manual of Statistics*
1910	Verrijn Stuart	*Inleiding tot de beoefening der statistiek*
1911	Yule	*An Introduction to the Theory of Statistics*
1912	King	*The Elements of Statistical Methods*
1915	Gini	*Appunti di Statistica*
1917	Mortara	*Elementi di statistica*
1917	Rugg	*Statistical Methods Applied to Education: A Textbook for Students of Education in the Quantitative Study of School Children*
1917	Secrist	*Introduction to Statistical Methods*
1920	Secrist	*Readings and Problems in Statistical Methods*
1920	Secrist	*Statistics in Business: Their Analysis, Charting and Use*
1921	Czuber	*Die Statistischen Forschungsmethoden*
1921	Jones	*A First Course in Statistics*
1921	Julin	*Principes de Statistique théorique et appliquée*
1922	Davies	*Introduction to Economic Statistics*
1923	Kelley	*Statistical Method*
1924	Jerome	*Statistical Method*
1924	Kent	*Elements of Statistics*
1924	Mills	*Statistical Methods Applied to Economics and Business*
1924	Riegel	*Elements of Business Statistics*
1925	Chaddock	*Principles and Methods of Statistics*
1925	Crum	*An Introduction to the Methods of Economic Statistics*
1925	Favett	*A First Course in Statistical Method*
1925	Fisher	*Statistical Methods for Research Workers*
1925	Gavett	*A First Course in Statistical Method*
1925	Niceforo	*La Methode Statistique et ses Applications aux sciences naturelles, aux sciences sociales et à l'art*
1925	Otis	*Statistical Method in Educational Measurements*
1925	Sutcliffe	*Elementary Statistical Methods*
1925	Thurstone	*Fundamentals of Statistics*
1925	Young	*Statistics as Applied to Business*
1926	Garrett	*Statistics in Psychology and Education*
1927	Bailey	*Statistics*
1927	Van Zanten	*Leerboek der Statistische Methode*
1928	Aftalion	*Cours de Statistique, professé en 1927-1928 à la faculté de Droit*
1928	Holzinger	*Statistical Methods for Students in Education*
1929	Banister	*Elementary Applications of Statistical Method*
1929	Florence	*The Statistical Method in Economics and Political Science*
1929	Lindquist	*Study Manual in Elementary Statistics*
1930	March	*Les principes de la méthode statistique*
1933	Davies	*Methods of Statistical Analysis in the Social Sciences*
1933	Rhodes	*Elementary Statistical Methods*
1934	Arkin	*An Outline of Statistical Methods (abbrev)*
1934	Richardson	*An Introduction to Statistical Analysis*
1935	Bayliss	*A Course in Business Statistics: The Elements of Statistical Methods (abbrev)*
1935	Fisher	*The Design of Experiments*
1935	Kramer	*A First Course in Educational Statistics*
1935	Moncetz	*Initiation aux methods de la statistique*
1935	Odell	*Statistical Method in Education*
1935	Richardson	*An Introduction to Statistical Analysis*
1936	Wheldon	*Business Statistics and Statistical Method*
1936	Goulden	*Methods of Statistical Analysis*
1937	Reebs	*The Elements of Statistical Method for Students in Elementary Education*

The text [31] by Arthur Lyon Bowley (1869–1957) is regarded by some as the first textbook on statistics though this may be unfair especially when compared with the text by Airy published 40 years earlier. Airy who was an astronomer encountered large volumes of data derived from stellar observations and would have been familiar with ‘measurement errors’ in both position and intensity. In contrast, Bowley was a lecturer in statistics at the London School of Economics and Politics (LSEP) during the years following its foundation in 1895, ultimately becoming the first Professor of Statistics in the U.K. when he was given a chair with that title at LSE in 1919. *The Elements of Statistics* [31] arose from his lectures and was conceived following his observation that there was ‘no textbook in English dealing directly and completely with the common methods of statistics.’ Bowley was a little dismissive of textbooks written in other languages.

Bowley's early editions of *The Elements of Statistics*, were essentially works in transition between the earlier commentaries on vital statistics and the later textbooks of statistical methods. The first three editions (1901, 1902, and 1907) are largely unchanged (xii + 336) and are divided into two parts with the first covering general elementary methods, whereas the second part is more theoretical, and covers the application of the theory of probability to statistics, especially the equation of the curve of errors, extension of the law of errors and its application, and the theory of correlation; the standard error is mentioned very briefly and the variance not at all. The fifth edition (1926), the closest in publication date to Woods and Russell, has the chapter on the application of averages broadened to include measures of dispersion, and the entire second part completely rewritten and expanded, and is more technical with its introduction of the calculus.

Bowley's second text [32], published in 1910, was *An Elementary Manual of Statistics*, essentially a more practically oriented version of his first text and ‘intended for those who desire some knowledge of statistical methods and statistical results without going deeply into technicalities or undertaking mathematical analysis.

By contrast to the two books by Bowley, George Udny Yule's 1911 book, *An Introduction to the Theory of Statistics* [33], is instantly recognizable as a text of statistical methods in the modern sense and, perhaps, has a better claim to be acknowledged as the first such book. The fifth edition (1919) acknowledges the help of ‘Captain M Greenwood of the Lister Institute’ as Yule was suffering from a severe eyesight impairment. The book was based on a one-year introductory lecture course on statistical methods given at University College, London, over the 8years from 1902 to 1909.

In general, the texts on statistics published up to 1937 can be regarded as of two types. The first is exemplified by Yule's book which provides a broad introduction to the subject as known at the time. The second is exemplified by Fisher's books, *Statistical Methods for Research Workers* [34] and *The Design of Experiments* [35]. These along with books by Davies and Crowder [36], Tippett [37], and Snedecor [38], the latter author having founded the Iowa State Statistical Laboratory [39], arose out of specific application areas. However, the much more broadly applicable methodological content of these latter books soon made them standard texts for anyone interested in statistical analysis.

It is clear, therefore, that by the mid 1920s there was a substantial body of published books that presented methods for the fundamental statistical aims of estimation and significance testing. By the early 1930s, statistics had developed further and was becoming characterized as an academic discipline [40], with 1933 being identified by Stigler [41] as the beginning of ‘Mathematical Statistics'.

Some of the specialized development of mathematical statistics would, we conjecture, have had limited direct relevance to medical statistics. However, Greenwood and Hill were clearly part of the more general statistics community as evidenced by their involvement with the Royal Statistical Society. In addition, from an unpublished memorandum [42], we know that Greenwood valued the role of the more mathematically trained statisticians.

An early influence on Greenwood was Karl Pearson, through Pearson's lectures at UCL It is unknown if Woods and Russell had any direct contact with Karl Pearson but, of Hill, Armitage [43] writes: ‘The young Bradford Hill, starting his work with Greenwood in 1923, also attended Pearson's lectures. He was less attracted than Greenwood to their mathematical content but equally spellbound by Pearson's personality’.

In terms of the influence of colleagues, Greenwood had appointed to his Unit, in 1931, Joseph Oscar Irwin (1898–1982) who graduated in Mathematics from Cambridge in 1921, after Irwin had spent time at both UCL and, with Fisher, at Rothamsted. Irwin stayed at LSHTM for 30years and is regarded as the leading theoretician among U.K. medical statisticians in the 1930s. Thus, through Irwin and otherwise, Hill, and Woods and Russell, would undoubtedly have been aware of the general developments, and publications, in statistics in the early part of the twentieth century. Whether specific publications were particularly in use at LSHTM, we do not know.

### 2.3. Biometry

The terms ‘biometry’ and ‘biometrics’ were also in use in the early part of the twentieth century and could be regarded as encompassing medical statistics; two texts can be highlighted.

Consider first the two editions of William Moses Feldman's (1879–1939) *Biomathematics: Being the Principles of Mathematics for Students of the Biological Science* [44]. The Preface to the first edition (1923) acknowledged Sir Walter M Fletcher (First Secretary, MRC), and Dr Major Greenwood and his colleagues in the Statistical Department of the Ministry of Health, especially for the chapter on biometrics which Feldman defined as the application of modern statistical methods to the measurements taken of biological (variable) objects; by contrast biomathematics was broader being defined as the science and art of rapid and accurate computation applied to the study and investigation of biological problems. The contents cover the basic material to the end of a first-year university course in mathematics but with a biological and medical slant in application. The content of the chapter on biometry might be considered a basic course in statistics (rather than specifically biometry) though the examples are drawn from demography and medicine. The book was later revised and expanded by Cedric Austen Bardell Smith (1917–2002) and ran to a further two editions (1954 and 1966); the chapter on biometry does not appear in the fourth edition.

Of more direct relevance to our discussion of Hill's work is Pearl's *Introduction to Medical Biometry and Statistics* [45] (three editions, 1923, 1930, and 1940 with 16, 17, 18 chapters, respectively; tables of contents are in our supplementary material).Pearl spent a year studying biometrics with Karl Pearson and was an original faculty member of Johns Hopkins University School of Hygiene and Public Health being the first professor and chairman of the Department of Biometry and Vital Statistics. His traditional work in vital statistics is reflected in his book *The Biology of Death* published in 1922 [46].

Pearl defines biometry as that branch of science which studies by methods of exact measurement on the one hand, and precise and refined mathematical analysis on the other hand, the *quantitative aspects of vital phenomena*. He argues that biometry deals with statistics derived from living things, or things that have at some time been living, and applies statistical methods, in the broadest sense, to such data.

In addition, Pearl stresses the importance of biometric ideas to medicine including an 1838 quote from *Jean Étienne Dominique Esquirol* (1772–1840). ‘Have they reflected that the sciences founded on observation can only be promoted by statistics? What is *experience* but the observation of facts, repeated often, and entrusted to memory? But the memory is sometimes treacherous; statistics register the facts, and forget nothing. Before a physician makes a *prognosis*, he has mentally calculated a probability, and resolved a problem in statistics; in other words, he has observed the same symptoms 10, 30, 100 times *(often)* in similar circumstances, whence he draws a conclusion. Every other mental combination deceives the practitioner. If medicine had not neglected this instrument, this means of progress, it would possess a greater number of positive truths, and stand less liable to the accusation of being a science of unfixed principles, vague and conjectural’.

It is easily seen that, in Pearl, Hill had a strong ally in his advocacy of careful statistical thinking in the medical sciences. It is not surprising therefore that there was extensive correspondence between these two individuals and we can therefore also infer that Hill's writing was done with the knowledge of Pearl's writings.

Finally, note that the term biometry today is being used in the context of biological means of identification of persons. However, the Biometric Society is attempting to ensure its continued use as it was conceived of in the 1930s and as it was characterized by Fisher at the time of the foundation of the Biometric Society: ‘Biometry, the active pursuit of biological knowledge by quantitative methods’.

With this background to the development of writings on medical statistics up to the 1930s, we now consider the two books emanating from LSHTM during this decade.

## 3. Woods and Russell

**Figure 1:**
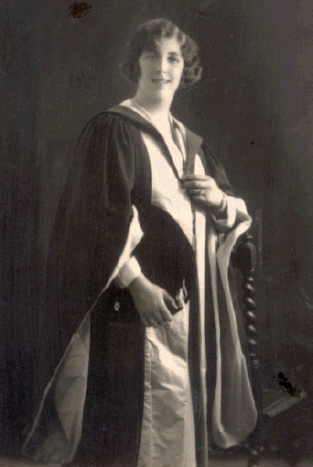
Portrait of Hilda Mary Woods in her University of London Doctorate of Science robes, May 1933. (Supplied and reproduced by kind permission of her niece (and adopted daughter) Rosemary Gear.)

Hilda Mary Woods and William Thomas Russell were two colleagues of Hill's at LSHTM. Hilda Woods was born in 1892 in Doddershall, Quainton, England, the daughter of William Ashburnham Woods and Mary Ann Woods (formerly Markham). She started government work in 1916 and at some stage was a member of the computing staff for the Ministry of Health Statistical Committee. Later she was appointed as an assistant lecturer in Greenwood's department at LSHTM. Woods was elected a fellow of the Royal Statistical Society in 1926, proposed by Greenwood and seconded by THC Stevenson, the Superintendent of Statistics at the General Register Office from 1909 to 1933. (He was awarded the Guy Medal in Gold from the Royal Statistical Society in 1920 and developed a social class classification into six ordered categories.) In May 1933 Hilda Woods was awarded a DSc by the University of London, titled ‘Vital Statistics’ and based on nine works on medical statistics and epidemiology (listed in our supplementary material). Figure 1 is a photograph taken in 1933 in her DSc academic gown. (Unfortunately we have been unable to locate a photograph of her co-author, William Russell.) Her examiners were Greenwood (internal) and Sir George Buchanan (1869–1936) who in 1934 was Master of the Society of Apothecaries of London and of whom Greenwood wrote an obituary for the *British Medical Journal*. On 17 November 1933, Woods sailed from London on the *Orama* (Orient line) which was bound for Australia; she disembarked at Colombo, Ceylon (now Sri Lanka), and in December married Roger Warburton Fowke. On 23 June 1936 she was awarded Membership of the Order of the British Empire (MBE, colonial office list) for social services in Ceylon. The membership lists of the Royal Statistical Society for 1946–1947, 1948–1949, and 1951, give her address in Nairobi, Kenya. She died on 29 November 1971, at Sandton, Transvaal, South Africa.

William Thomas Russell was born in Killarney, Ireland, the son of a schoolmaster, and educated in Dublin. He moved to England before World War I and after a period of commercial employment, in 1915 he joined the staff of the Medical Research Committee. He worked first with John Brownlee (until 1927) mainly at the National Institute for Medical Research, Hampstead, London, and later with Greenwood (until 1943) at LSHTM. He lectured in the introductory statistics course but left LSHTM in 1943 on appointment as statistician in the newly formed Institute of Social Medicine in Oxford under John Ryle. He worked there until his death in 1953. His obituary [47] records that he wrote many sound papers in the field of vital statistics, including a study on the epidemiology of diphtheria conducted in England from 1901 to 1940, the effects of fog and cold on mortality from respiratory disease, geographical and secular patterns of tuberculosis, the sex-ratio at birth, and appendicitis. He was elected a fellow of the Royal Statistical Society in 1930 and in 1943 was awarded a DSc by the University of London for work on epidemiology and demography, and based on 25 publications (listed in our supplementary material). Sutherland [47] recalls that Russell, like Hill [43], ‘… always preferred a direct approach and a simple table to more refined statistical techniques, which he often felt to be unnecessary sophistication’.

## 4. *An Introduction to Medical Statistics* by Woods and Russell

In 1931, the first edition of Woods' and Russell's *An Introduction to Medical Statistics* [4] appeared. It was published by PS King and Son Ltd., Orchard House, Westminster (and printed in Great Britain by Richard Clay and Sons Ltd., Bungay, Suffolk). A second edition followed in 1936 from the same publisher (but printed by Phototype Ltd., in Barnet, Hertfordshire). The second edition was republished in 1948 by Staples Press (Cavendish Place, London, and printed by them at Great Titchfield Street, London).

The contents (11 chapters) and pagination of the two editions are almost identical. A line-by-line comparison of the first and second (reprinted) editions reveals very few differences between them (correction of two figures in the table on page 45, some changes to digits after the decimal point (pages 61, 63, 64, 86, 92, 93), and minor word changes (diagram V, pages 51, 61), or small additions (page 105). The book has a preface by Greenwood who states that ‘the object of this little book is to help a student acquire facility and confidence in carrying out the simpler operations of statistical analysis’; he continues ‘For the general reader who wishes to understand economic and social statistics there are plenty of guides. But the statistics of the General Register Office are quite as important as those of the Board of Trade or the Ministry of Labour to anyone who wishes to understand the world we live in, and guides to the methods used in analyzing them are not so easy to find’, a view with which we concur given our review. The book has no index or acknowledgements though it has textual references to two books on vital statistics [27, 28], and one paper by Woods on construction of life tables [48]. The only prefatory comment by the authors is a statement that ‘this book is intended only as a simple introduction to statistical analysis suitable to the needs of those taking the course for the Diploma in Public Health’ presumably at LSHTM, and those who desire to study the subject further are recommended to read Yule [33] and Pearl [45]. At the end of the first edition there is a list of ‘books to read’ in the form of an advertisement and this includes the two statistics books by Bowley (fifth and fourth editions), as well as his book with Hogg *Has Poverty Diminished?*, followed by other books on public health, health administration, or social conditions.

We are aware of two published reviews of the first edition of Woods and Russell, one in the U.K. and one in the U.S.A. The first published in the *Journal of the Royal Statistical Society* in 1931 was by G. U. Y. (clearly George Udny Yule (1871–1951)) [49]. Yule quotes from Greenwood's introductory preface explaining that the book is intended to help the student acquire the facility and confidence to carry out the simpler operations of statistical analysis. Further, he suggests that any student who has worked through the course at LSHTM that the book is intended for should be prepared to go further and tackle books on pure vital statistics such as Newsholme and Whipple or even Pearl's book on biometry. Yule ‘found little to criticize’ and confined his comments to the retention of fewer figures in some worked examples, the reason against the choice of the 1901 population as a standard, and sundry printer's errors. He also made a plea for the inclusion of a chapter on the trustworthiness of data, on the difficulties of comparison due to continually changing classifications both in census and registration data, to improvements in diagnosis, and so forth.

In contrast, the other reviewer, E(dwin) W. Kopf (1888–1933) is scathing [50] and declares ‘the authors have failed definitely to accomplish the purpose they had in view’, with explanations that are ‘vague and misleading.’ He notes that ‘*de facto*’ (actually resident) and ‘*de jure*’ (legally resident) populations are discussed without clear definition; the passage on quantitative data is meaningless; confusion between fertility and fecundity; that the definition of infant mortality is clearly wrong; lack of agreement between the definition of attack rate and the example; and then broadens his criticism to whole chapters—those on averages, measures of dispersion and correlation ‘will certainly mislead students,’ that on life tables is ‘badly confused,’ and the chapter on sampling ‘seems nowhere to be suited to the needs of students examining quantitative data on pathological phenomena.’ His final shot is the lack of a definition of medical statistics; ‘it seems impossible to impart instruction in a subject without telling the students what the discussion is about’. Kopf worked for the Metropolitan Life Insurance Company in New York, he was a Fellow of the American Public Health Association, and a Fellow of, and Chair of, the Educational Committee of the Casualty Actuarial Society (in 1931). He wrote about the statistical work of Florence Nightingale (*Journal of the American Statistical Association* 1916:15;388–404), a statistical study of the influenza epidemic (1919), and about the origin and development of reinsurance (1929), and co-authored a paper (with Fales and Tobey) on vital statistics: constitutional, statutory, and administrative aspects, and a book (with Dublin and Van Buren) *Mortality Statistics of Insured Wage-earners* (1919); he was also a learned discussant of a paper on an analysis of the death rate of Detroit by Deacon. Kopf was clearly an expert on both vital and actuarial statistics, as was Yule, and it is perplexing to see such polarized views. However, some of Kopf’s criticisms are not specific and our reading of relevant passages of the book suggest that they may be slightly pedantic.

In 1948, there was a further very brief review [51] of the 1948 reprint of the second edition of Woods and Russell in the *British Journal of Industrial Medicine*, which followed a review of the fourth edition of Hill. The book is described as ‘more elementary than Professor Bradford Hill's’ but ‘also a very useful introduction to medical statistics, suited to the needs of people studying for the Diploma of Public Health.’ In addition, the reviewer indicates that ‘the exposition throughout maintains a high standard of clarity.'

When Hill was writing his articles for *The Lancet*, Woods and Russell's book would likely have been in use as a text for teaching at the LSHTM, and therefore very familiar to him. Indeed, Hill's first edition has an acknowledgement to Russell in the preface, a full reference to Woods and Russell in the text on page 118 and an abbreviated reference on page 147. In addition, there is a further reference to Woods and Russell, for details of a calculation, in the Lancet version of Hill's chapter 4 which was not retained in the book when the last two Lancet articles, written after the others to deal with specific calculations, were incorporated as earlier chapters in the book. Further, in the second edition of Hill, one of the two references to Woods and Russell in the first edition was removed when a chapter on the calculation of standardized death-rates was included by Hill.

The aim of Hill's writing must therefore have been different from that of Woods and Russell. In a foreword to Hill's book, the Editor of the Lancet indicates one motivation when writing We have reason to believe that there is now a steadily increasing demand among both clinical and public health workers for some knowledge of that [statistical] technique and a realization that it is not much good collecting figures more or less haphazardly and then to expect a professional statistician to draw conclusions from them'. It is interesting therefore to examine Hill's writing through a comparison with that of Woods and Russell. More generally, as indicated in the introduction, we want to examine what differentiated the two books and why Hill's book made the impact it did.

## 5. Comparison of the content of Woods and Russell and Hill

As illustrated in Table III, a scan of the contents of Woods and Russell and then of Hill reveals a significant overlap in the topics covered. This is more clearly revealed in Table IV where the various sections of Woods and Russell are matched by topic with the chapters of Hill. It can be seen that almost every topic in Woods and Russell, with the exception of vital statistics, is also dealt with by Hill. Although Hill does contain some additional material, notably in the first two chapters and the last, the primary distinction between the two books lies, therefore, not in the topics covered but in the manner in which they were presented. To consider this further, we have made a comparison of five specific topics, tables and charts, standardized rates and life tables, measures of dispersion, correlation and regression, and sampling.

**Table III:** Titles of chapters in Woods and Russell, and Hill.

Chapter	Woods and Russell (1931) *An Introduction to Medical Statistics*	Hill (1937) *Principles of Medical Statistics*
I	Vital statistics	The aim of the statistical method
II	Tabulation of data	Selection
III	Construction of charts and graphs	Presentation of statistics
IV	Population	The variability of observations
V	Standardized death rate	Calculation of the standard deviation
VI	Averages	Problems of sampling: averages
VII	Measures of dispersion	Further problems of sampling: proportions
VIII	Correlation	Further problems of sampling: differences
IX	Coefficient of regression	Further problems of sampling: *X*^2^
X	Life tables	Further examples and discussion of *X*^2^
XI	Sampling	The coefficient of correlation
XII	—	Calculation of the correlation coefficient
XIII	—	Life tables and survival after treatment
XIV	—	Common fallacies and difficulties
XV	—	Further fallacies and difficulties
XVI	—	Further fallacies and difficulties
XVII	—	General summary and conclusions

**Table IV:** Contents of Woods and Russell matched with Hill (first edition).

	Woods and Russell (1931) *An Introduction to Medical Statistics*	Hill (1937) *Principles of Medical Statistics*
Chapter	Title (Sub-headings from Contents)	Chapter
I	Vital statistics	—
	The census	—
	Registration of births and deaths	—
	Registration of live births	—
	Registration of deaths	—
	Still-births	—
	Registration of sickness	—
II	Tabulation of data	III
III	Construction of charts and graphs	III
IV	Population	
	Estimates: Arithmetic	VI
	Estimates: Geometric	—
	Birth-rate	—
	Death-rates	XVI
	Infant mortality	XII, XIV
	Specific death-rates	—
	Proportional mortality	XV
	Occupational mortality	XV
V	Standardized death rate	XVI
	Direct method	XVI
	Indirect method	XVI
	Occupational mortality	XV
VI	Averages	VI
	Arithmetic mean	VI
	Median	—
	Mode	—
	Frequency distribution	III
VII	Measures of dispersion	IV, V
	Range	IV
	Mean deviation	IV
	Standard deviation	IV, V
	Standard deviation of ungrouped series	V
	Standard deviation of grouped series	V
	Coefficient of variation	IV
VIII	Correlation	XI
	Coefficient of correlation	XI
	Coefficient of correlation for ungrouped data	XII
	Coefficient of correlation for grouped data	XII
X	Coefficient of regression	XI
XI	Sampling	VI, VII, VIII
	Sampling of attributes	IX, X
	Sampling of variables	—

### 5.1. Tables and charts (Woods and Russell, chapters II, III; Hill, chapter III)

Woods and Russell introduce their recommendations for data presentation by pointing out that, once collected, data must be arranged in ‘some kind of tabular form so that particular factors may be easily studied and compared.’ They give some simple examples and describe two practical methods for counting—cards sorted into heaps, and the system of tallying 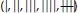. Both before, and for many years after 1930s, such work was tedious, and any aide to simplify and speed up counting was welcome.

The following chapter, devoted to the construction of charts (or graphs), points out that these may be a step towards even greater clarity than can be achieved with tables provided they are clearly constructed without ‘too much information', a point re-emphasized and beautifully illustrated by Tufte [52] over 50years later. Woods and Russell mention three types of graphs: line charts, bar-charts, and histograms. Apart from specific instructions on how to construct charts, they emphasize the importance of adequate labelling so that reference to the text is not required; choice of scales that do not distort differences; use of vertical scales that start at zero and the need to distinguish, and provide a key for, multiple lines within the same figure. Their final chart (produced on a double-page foldout) shows weekly notifications of five different diseases in London during 1929; the five diseases are all measured from the same zero base so that the chart is actually five superimposed histograms. The chapter ends abruptly at this point with no discussion of the interesting conclusions that might be drawn from such a figure.

In contrast to Woods and Russell's two chapters occupying 18 pages, Hill's discussion of presentation is confined to a single chapter of 10 pages that nonetheless follows the same pattern of discussing tables and graphs separately. From the start Hill emphasizes the importance of first determining the questions that the data are capable of answering, and then deciding on the form of the presentation that brings out the answers most clearly. His important, underlying message, is the construction of the frequency distribution.

Hill then sets out some basic principles for the presentation of statistical data that extend beyond those of Woods and Russell. The section ends with a quotation from Pearl (second edition), who recommends scrutiny of published tables, and asking, ‘What is the purpose of this table? What is it supposed to accomplish in the mind of the reader?…. Wherein does its failure of attainment fall?', as well as advice to look at the *Annual Reports of the Registrar-General for England and Wales*.

Hill's section on graphs discusses five points: need clarity; distortion from expansion or compression of scales, including non-zeroed scales (illustrated by death rates from cancer over 30years from 1901); regarding them as subsidiary aids, not as *the evidence*; proper choice of vertical scale to illustrate absolute and relative changes (well illustrated by death rates per million from respiratory tuberculosis and typhoid/paratyphoid fever over the period from 1870 to 1930, also plotted as percentages of the numbers in 1870s); and finally, labelling to make them self-contained. Hill is adamant that both tables and figures are necessary; ‘It is the essence of science to disclose both the data upon which a conclusion is based and the methods by which the conclusion is attained’, and his advice to editorial pleading of the sort ‘if we print the graphs would it not be possible to take some of the tables for granted?’, is partly to turn a deaf ear, and partly to point out that ‘statistical tables are not a step to a diagram, they are the basic data’!

For this topic, a fair conclusion is that Woods and Russell devoted more attention to the mechanics of table and graph preparation than Hill but less to the value and use of these forms of data presentation. While the latter was not ignored by Woods and Russell, Hill put it at the forefront of his presentation.

### 5.2. Standardized rates and life tables (Woods and Russell, chapters V, X; Hill, chapters XIII, XV, XVI)

#### 5.2.1. Standardized rates

Standardized death rates and life tables represent familiar topics in the context of vital statistics. At the time Woods and Russell was written, chapters on these topics would therefore have been very natural. Standardized death rates are treated first in Woods and Russell, in the fifth chapter with data on the mortality of railwaymen and clergymen from 1900 to 1902 used for illustration. The two primary methods of standardization, direct and indirect, are then introduced with the direct methods stated to be better and always recommended for use if age-specific death rates in the population of interest are available.

If the directly standardized rates are to be used for comparison purposes, Woods and Russell suggest that we ‘reduce our standard population to a million’ which avoids some extra calculations. The section concludes with a paragraph beginning ‘It will be clear from what has been said that the choice of a standard population is not a matter of complete indifference. A standardized rate of mortality is a weighted average’. The potential for very extreme bias is then discussed but the paragraph concludes ‘In practice, no sensible bias of this kind would arise unless the age composition of the standard population was so very bizarre that nobody would be likely to accept it as a reasonable one’. In general, the discussion of directly standardized rates is very consistent with modern treatments.

In contrast, modern treatments often focus on the standardized mortality ratio (SMR) when discussing indirectly standardized rates. Woods and Russell show how to work out the expected number of deaths in the study population if the age-specific rates of the standard population were acting. However, this is then divided by the total study population size to get a so-called ‘index death-rate.’ This then corresponds to the crude rate that would be observed if the standard population's mortality rates were acting and this is compared with the observed crude rate in the standard population to produce a ‘standardizing factor’ defined by the ratio


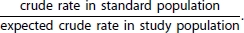


The crude death rate in the study population is then multiplied by this standardizing factor to generate an adjusted crude rate for the study population which takes into account its favourable or unfavourable age and sex distribution relative to the standard population. The presumption appears to be that this adjusted crude rate will be used for comparisons with other areas. This indirectly adjusted rate would now more commonly be defined as the product of the SMR, the ratio of observed to expected deaths in the study population, and the crude rate for the standard population but much more use would be expected to be made of the SMR itself.

Hill has a much shorter discussion of standardized mortality rates. The problems with the comparison of crude rates are pointed out in 1½ pages and the basic approach to the calculation of a directly standardized rate is outlined in another page. Reference is then made to Woods and Russell for complete details and discussion of indirectly standardized rates. The brief section finishes with the questions related to age/sex distribution that one should ask when considering published crude rates. The final sentence is ‘Crude rates themselves should never be accepted without careful consideration on those lines.’

##### 5.2.1.1. Life tables

The start of the chapter on life tables in Woods and Russell is perhaps reflective of the general style of the book. ‘Although the importance of expressing mortality data in life-table form can be exaggerated, nevertheless, the use of life tables in affording summary comparisons of place with place or epoch with epoch is considerable. Life-table construction, however, has never been very popular with Medical Officers of Health, simply because they have been rather daunted by the mathematical appearance of the method used in their construction. But the employment of mathematical formulae is not a vital necessity’.

After this introduction, Woods and Russell describe the classic life table construction based on longitudinal follow-up of individuals, focusing on the calculation of expected additional years of life given that an individual has lived *x* years. The assumption that individuals live a ½ year in the year of their death is recommended. They then point out that this type of calculation is often not possible and they present an alternative, taken from a paper by Woods [48]. The formula given for the estimated probability of dying between *x* and *x* + 1 is: *2m_x_*/2 + *m_x_*, where *m_x_* is the death rate for the *x*th year of life. It is obtained by dividing the number of deaths between *x* and *x* + 1 by the population living at the middle of the year. Woods and Russell then point out that this can be simplified as


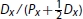


where *D_x_* is simply the number of deaths in the year of age *x* to *x* + 1. *P_x_* is the census enumeration of the number of persons who were aged *x* at their last birthday. Excepting years under age five, when census enumeration was felt to be weak, such rates can be used to create official life tables, an example of which is given.

As for standardized death rates, Hill references Woods and Russell for details on the calculation of life tables. However, his Chapter XIII begins with the presentation of the ‘English Life Table’ and explains what it represents. The probabilities *q_x_*, corresponding to the ‘probability, or chance, of dying between age *x* and *x* + 1’, are highlighted. Interpretation of ‘The National Life Table’ is emphasized, as showing ‘how a population would die out if it experienced as it passed through life the same death-rates as were prevailing in England and Wales in 1930–1932’. This emphasis is not found in Woods and Russell.

The calculation of a life table based on longitudinal data, which begins the chapter on life tables in Woods and Russell, is dealt with in Hill in the context of measuring survival rates after treatment, a subject not addressed in Woods and Russell. The essential structure of conditional probabilities for each additional year of survival is characterized and the use of these to calculate a survival curve is recorded in a column labelled ‘Number Alive on Each Anniversary out of 1000 Patients’, with the radix 1000 chosen for convenience. Hill also deals with the exclusion of patients or censoring and thus provides the basic structure, now identified with Kaplan and Meier, hence widely used in medical statistics.

An examination of the treatment of standardized rates and life tables thus shows clearly that Woods and Russell were writing in the light of their contact with work in vital statistics. They are concerned to make the needed calculations clear while also commenting on their appropriate use. Hill evidenced little interest in the details of the calculations but dealt almost exclusively with interpretation and usefulness. More importantly, we see that some of the examples used by Hill emphasize, not traditional vital statistics, but rather questions that arise in medical statistics such as patient survival and epidemiological risk factors.

### 5.3. Measures of dispersion (Woods and Russell, chapter VII; Hill, chapters IV, V)

Woods and Russell introduce the importance of assessing the extent to which observations are scattered about the mean with an example based on 10 observations of heights measured in each of two groups of boys. They then ask how this variation or scatter can be measured, and discuss four parameters—the range, mean deviation, standard deviation (SD), and coefficient of variation. Although the frequency distribution is referred to in both earlier (VI. Averages) and later (XI. Sampling) chapters, there is no explicit mention of it here, and no figure to illustrate the concept of variation. The range and mean deviation are discussed (and dismissed) briefly with calculation of the latter set out in an example of examination marks obtained by five boys; the SD, and its calculation, occupy most of the chapter. The chapter ends with a brief discussion of the coefficient of variation, introduced as a technique for comparing the relative degree of variation in two distributions that are measured in different *kinds* of units; the example uses heights and weights in girls.

In contrast, Hill starts with the frequency distribution, stresses the limitation of the mean by itself, and points out that variability (or lack of it) is an important characteristic. This is immediately illustrated by ages at death from diseases of the Fallopian tube (206 deaths), and abortion (99 deaths) in 1934; the frequency distributions are presented in *both* a table *and* a figure. Of note is that Hill uses examples with comparatively large samples that are of immediate relevance to medicine rather than the much smaller samples of ‘more remote’ demographic data for height and weight employed by Woods and Russell. He discusses the *range, mean deviation*, and SD in the context of a sample of 20 observations of systolic blood pressure; his critique of the first two is more astute than that of Woods and Russell as he points out that one, derived solely from the two most extreme observations, ignores the spread of all observations within these limits, whereas the other is limited by statistical problems in *sampling*. Interpretation of the SD is illustrated by the two distributions of age at death and for these, Hill points out the importance of the SD in assessing whether the differences between two means, or two SDs, have arisen by chance. The final measure, the coefficient of variation, is presented in a similar context to Woods and Russell.

Towards the end of this chapter Hill again emphasizes the importance of variability, reproducing Yule's advice to ‘get out of the habit of thinking in terms of the average, and think in terms of the frequency distribution,’ and follows this with further examples of variability, some discussion of symmetry, and the usefulness of a figure showing the frequency distribution.

The following chapter (V) sets out methods for the calculation of the SD for both ungrouped and grouped series of observations, using the methods described by Woods and Russell. However, Hill also presents methods for checking the arithmetic, and mentions use of the divisor *n* − 1, instead of *n*, in small samples.

This topic therefore again illustrates the much broader view of a topic which characterizes Hill's presentation. In this case, he does provide the calculation details and one might speculate that this is because it is such an important topic and fundamental to many of the statistical methods in use at the time.

### 5.4. Correlation and regression (Woods and Russell, chapters VIII, IX; Hill, chapters XI, XII)

Woods and Russell indicate that the problem to be considered is ‘the measurement of the degree of correlation or association between two variables. We need to know if large values of the one variable coexist with large values of the other’. Interestingly, they mention that in ‘the field of vital statistics’ there are numerous examples of this type of problem and give examples. This raises the question of whether they are distinguishing between vital statistics and medical statistics.

Two examples are used to establish the existence of associations and then they turn to the correlation coefficient as a ‘quantitative measure of the degree of association’ with a brief description, a couple of paragraphs, of the correlation coefficient in words. It is described as measuring how ‘observations deviate conjointly from their means’ and that ‘the correlation coefficient is really a measurement of individual variability in terms of average variability’. The range, −1 to 1, is discussed.

They describe in detail the calculation of the correlation coefficient for ungrouped and grouped data. For the former, the example is of the link between poverty, measured as the percentage living more than two in a room in London Boroughs, and diphtheria rates. The grouped data is of age and weight of school boys. (Note that this example is mentioned briefly in Hill when discussing that regression lines should not be extended; page 105).

Woods and Russell then have a separate chapter on the regression coefficient with a good introductory paragraph suggesting that it may be more useful than the correlation coefficient and giving the origin of the term regression from Galton's study of heights of sons and fathers. (The origin of the term does not appear to be in Hill).

The calculation formula for the regression coefficient is given simply in terms of the correlation coefficient and the standard deviations of the variables and the existence of two regression coefficients is mentioned with a comment that ‘We compute the one which suits our purpose best’. The example used for illustration is a regression of weight on age, using the same data as used in the previous chapter.

Very limited mention of variability is made in Woods and Russell and this provides the most obvious contrast with Hill, whose Chapter XI describes the problem of ‘the measurement of the *degree* of relationship between two, or more, charateristics of a population’. His introductory example is of the relationship between deaths due to bronchitis and pneumonia and mean temperature. Immediately, Hill discusses the variability in the number of deaths registered in weeks with the same mean temperature and presents a scatter plot with a straight line relationship also drawn; the choice of the line is not described. He indicates how the correlation coefficient is a ‘satisfactory measure’ if a relationship is adequately represented by a straight line with ‘no tendency to be curved’.

After discussion of some examples and of mean square deviations, there is a sub-section specifying ‘The Regression Equation,’ the last paragraph of which describes the slope value as a regression coefficient and the equation as a regression equation, and finishes with a summary of what has been learned about the example so far.

Hill then discusses ‘Precautions in Use and Interpretation’ of the correlation coefficient in four sub-sections: the relationship must be representable by a straight line, the line must not be unduly extended, association is not necessarily causation, and the standard error. The last sub-section gives a formula, 1/square root(*n*−1), for the standard error and shows how to carry out a test of significance which ‘should be applied before any attempt is made to interpret’ a correlation coefficient. In a brief summary section, Hill mentions the possibility of multiple linear regression but ‘the methods are beyond the limited scope of this book’. After this chapter dealing with the use and interpretation of correlation coefficients and regression lines, the next chapter deals with the details of calculation.

Woods and Russell provide a good account of correlation and regression, their purposes and interrelationship. One assumes that therefore that this was an established topic in the courses at LSHTM at the time. It is difficult to see why they did not address the issue of variability since, as Hill did emphasize, this is critical for the use of this methodology. Also, again, Hill, for this topic, chose examples that would engage the attention of medical researchers and illustrate the potential value of the methods in areas where it may have been applied infrequently.

### 5.5. Sampling (Woods and Russell, chapter XI; Hill, chapter VI, VII, VIII, IX)

Much of what is labelled ‘sampling,’ in Woods and Russell and in Hill, deals with what would now be termed statistical inference. We have argued that Hill was conscious of this aspect of medical statistics throughout his writing whereas it received little attention from Woods and Russell. However, in their last chapter, entitled *Sampling*, Woods and Russell discuss significance levels in a very enlightened manner, as highlighted by Vandenbroucke [53] in one of the rare recent references to Woods and Russell.

In that chapter, Woods and Russell make two important points. The first is that a very small significance level still presents a choice between two alternatives, the rejection of the null hypothesis or that a rare event has occurred. While the former may often result, Woods and Russell give the hypothetical example of a clinical trial of coloured water as treatment for typhoid. They argue that, in such a case, the probability of a rare event is still greater than that the null hypothesis of no effect due to coloured water is false. The second point is that any choice of a cut-off level to determine significance is ‘quite arbitrary.’ Woods and Russell write ‘It is much better in any important case to state the arithmetical facts as revealed by the SD’.

The contrast with *Principles of Medical Statistics* is noteworthy here in that Hill fairly consistently writes of simple yes/no decisions regarding ‘significance’.

### 5.6. A summary comparison

A detailed look at Woods and Russell shows that it has clear links to the nineteenth and early twentieth centuries, the era of vital statistics, and the associated texts. It does build in some areas on this work and introduces topics of fundamental importance to medical statistics more generally. The primary focus of the book can properly be regarded to be on calculations although it goes beyond being simply a computational handbook since there is some attention to interpretation, applicability and, in the last chapter, to statistical inference.

In many ways, Woods and Russell is a very attractive book. It provides a good introduction to medical statistics as it was developed from work in vital statistics. Vandenbroucke [54] states that ‘it was almost forgotten’ but was clearly impressed remarking that ‘in this book many of the emphases were truly ‘Bayesian avant la lettre’ and had, in contrast to later statistical writings, little regard for p values.’ It could be debated whether Woods and Russell might better be described as ‘Fisherian,’ and their discussion of p-values is primarily limited to their last chapter, but these are minor quibbles.

In contrast, Hill has little interest in the extensive past work on vital statistics and his book looks forward to the modern era. As discussed earlier and as indicated in the preface, the book was heavily influenced by Hill's ongoing research activities. He was also, clearly, well aware of the statistical methodology which had been and was being developed. While his purpose was not to discuss this comprehensively, concepts such as that of significance testing arose naturally throughout his writing, and through his few references to Woods and Russell, we conclude that he regarded it as a computational reference and did not want to emulate their goals. Nevertheless, it is remarkable that the two books originated from the same department and it is reasonable to conclude that Hill must have been influenced to some extent by Woods and Russell.

Woods and Russell can be regarded as written, at least in concept, for the students at LSHTM who would need to know the material presented and who would be aiming to apply it. Hill, in contrast, had a much broader goal. The content of his articles, their simple style, comparative brevity, clarity of exposition, and practical nature, rendered them and the subsequent book of immediate usefulness to those wanting to study the comparatively new discipline of medical statistics. More importantly, it would increase the number of such people by demonstrating the potential impact of the new discipline.

## 6. *Principles of Medical Statistics* by Austin Bradford Hill

### 6.1. The beginning

The preceding material has indicated that Hill's book was both innovative and useful to a wide range of readers. And, as indicated earlier, the invitation to Hill to write *The Lancet* articles came from Pamela Kettle, an assistant editor of the journal. But what was the genesis of the book itself?

Hill's correspondence concerning the book can be found in the collection of letters held in the MRC Biostatistics Unit. From a later letter dated 26 January 1937, we learn that, at some stage in 1936, Hill made an ‘only half-serious request’ to consider publication of the articles as a book. This suggestion was encouraged by Sir Squire Sprigge, Editor of *The Lancet* (letter dated 16 December 1936) who believed that *The Lancet's* publishers ‘would probably take the book at once’ and was prepared to tell them that ‘the subject is one that particularly requires some fundamental elucidation’. However, 2 months later there were reservations with F.G.H. Holt (Secretary at *The Lancet*, letter dated 25 February 1937) reporting that the Directors (presumably of the journal's Management Company) were ‘apprehensive of the production and success of a small book at a low selling price’. Hill's response was immediate (26 February 1937):

I should like to know as early as possible whether the Directors are willing to publish my articles in book form. I certainly understood from my correspondence with the Editor that that was the intention and I took a very great deal of trouble over the articles with that end in view. (A number of people have expressed to me the hope that the articles will be put into a book). If the Directors however feel that its success is too speculative I should wish to approach another publisher.

(It is not known if this was the exact content of the letter that was sent; the above is a draft written at the foot of the letter from F.G.H. Holt who refers to it in his subsequent letter of 5 April 1937.)

After another 2 months *The Lancet's* Management Committee relented though with reservations and conditions (letter from F. G. H. Holt dated 5 April 1937), as ‘it was considered improbable that financial success would be achieved, and inferred that a period of time would elapse before even the cost of production could be recovered, apart from the substantial expense of publicity’; the conditions amounted to an estimated number of between 500 and 1000 copies being free of royalties. Hill modified these terms (letter dated 9 April 1937) preferring a fixed figure to one based upon an ‘approximate estimate of the cost of production'; he suggested foregoing royalties on the first 500 copies, then 10 per cent of the published price on (ordinary) sales from 500 to 1500 copies, of 12½ per cent from 1500 to 2000 copies and 15 per cent on all subsequent sales. *The Lancet* agreed to these terms despite the cost coming out ‘more than I expected’ (letter from F.G.H. Holt dated 26 April 1937).

The cautions of *The Lancet* management were somewhat misguided in retrospect. Pearl was far more prescient and wrote to Hill (letter dated 5 May 1937) ‘in my opinion they (*The Lancet* people) are quite needlessly worried about the extent of its sale. Your presentation is excellent and I am sure the book will have a steady sale for a long time’.

### 6.2. The editions

Pearl's view was shared by Fisher who wrote to Hill on 9 April 1937 saying, ‘I am very glad to hear about your book. It, and probably others like it, are certainly much needed’. And empirical confirmation of this view was provided when, only 2years after the publication of the first edition, the second edition was launched.

The last edition, of the 12 published, to retain the original name was the 9th, published in 1971. In his preface, Hill reiterates, from his preface to the sixth edition, that contemporary examples are not necessary for illustrative examples ‘but possibly it may appear to some readers to be important, and an air of modernity can at least do no harm’. However, he remained keen not to change what had been successful and therefore ‘decided to leave alone much that seemed well.’

In our supplementary material we provide a table that tracks the publication history of Hill's book. The table is sufficient, in itself, to indicate the special place this work has in the history of medical statistics.

Another study would be required to track all the changes in the various editions, and may offer further revelations on the subsequent development of medical statistics more generally. Some comparisons were made by Horton [55] who felt that ‘the changes he made to his text provide a valuable insight into the evolution of medical statistical thinking during the course of this century’. Chalmers [56] has also carried out a comparison related to the allocation of treatments in clinical trials and tracked the changes from alternation to randomization from the first to the ninth editions.

Yet another study could examine texts that appeared subsequently starting in the next year, 1938, with Mainland's *The Treatment of Clinical and Laboratory Data: an Introduction to Statistical Ideas and Methods for Medical and Dental Workers* [57], which acknowledges both Pearl and Fisher; it is a basic textbook of statistical methods packed with illustrative examples and worked calculations. Its primary objective is to present ideas and some methods that may help the medical or dental clinician who wishes to benefit from his own observation and from a critical reading of statements made by others.

## 7. Conclusion

We have considered the background to and the writing of two early texts on medical statistics. From the retrospective perspective we now have, Hill's book stands out as making a worldwide contribution to the understanding and teaching of medical statistics over the last 70years. It remains the best known text on the subject.

In contrast to Hill's *Principles of Medical Statistics*, Wood's and Russell's *An Introduction to Medical Statistics* is comparatively unknown. However, it is no disservice to Hill to bring this lesser known volume out of obscurity since it deserves to be recognized as reflecting an important stage in the transition from vital statistics to medical statistics. Indeed, although limited in its content, it can be regarded as in the forefront of texts on medical statistics as the subject is now understood. Given the background of the two authors, this is a particularly considerable achievement. Their ability to move from relatively routine vital statistics to broader interests was fostered in the environment of the LSHTM and by Greenwood's leadership of the work there. But as in all such situations, the individuals involved had to take advantage of the opportunity and their work is a testament to their willingness to take on what must have been a particular challenge.

## Supplemental material

Additional background information is available at http://www.mrc-bsu.cam.ac.uk. This includes an expanded version of Table II; the contents of the three editions of the book by Pearl; the publications submitted for the degree of DSc, together with other publications, for both Hilda Mary Woods, and William Thomas Russell; and, a publication history of the 12 editions of Hill's book and of his original Lancet articles.
