# Supplementary material for: Woods and Russell, Hill, and the emergence of medical statistics
Source: Stat Med. 2010 May 14;29(14):1459–76. doi: 10.1002/sim.3893 (PMC2991772; doi:10.1002/sim.3893)
Supplement: Supplementary file 1 [file sim0029-1459-SD1.pdf]

Supplementary Material

Woods and Russell, Hill, and the Emergence of Medical Statistics

Vern Farewell and Tony Johnson

Table S1: The Timeline of Textbooks on Statistics to 1937 (full)

Year	Author	Title and Edition
1861	Airy	1st ed. *On the Algebraical and Numerical Theory of Errors (abbrev.)*
1875	Airy	2nd ed. *On the Algebraical and Numerical Theory of Errors (abbrev.)*
1879	Airy	3rd ed. *On the Algebraical and Numerical Theory of Errors (abbrev.)*
1886	Meitzen	*Geschichte, Theorie, und Technik der Statistik*
1897	Thiele	*Elementǽr Iagttagelseslǽre*
1889	Thiele	*Forelæsninger over Almindelig Iagttagelseslære*
1890	Westergaard	*Gnendzüge der Theorie der Statistik*
1899	Davenport	1st ed. *Statistical Methods with Special Reference to Biological Variations*
1901	Bowley	1st ed. *Elements of Statistics*
1902	Bowley	2nd ed. *Elements of Statistics*
1903	Thiele	*Theory of Observations*
1904	Davenport	2nd ed. *Statistical Methods with Special Reference to Biological Variations*
1906	Elderton	1st ed. *Frequency Curves and Correlation*
1907	Bowley	3rd ed. *Elements of Statistics*
1909	Elderton	1st ed. *Primer of Statistics*
1909	Thiele	*Interpolationsrechnung*
1910	Bowley	1st ed. *An Elementary Manual of Statistics*
1910	Elderton	2nd ed. *Primer of Statistics*
1910	Verrijn Stuart	1st ed. *Inleiding tot de beoefening der statistiek*
1911	Yule	1st ed. *An Introduction to the Theory of Statistics*
1912	Elderton	3rd ed. *Primer of Statistics*
1912	King	1st ed. *The Elements of Statistical Methods*
1912	Yule	2nd ed. *An Introduction to the Theory of Statistics*
1914	Davenport	3rd ed. *Statistical Methods with Special Reference to Biological Variations*
1915	Bowley	2nd ed. *An Elementary Manual of Statistics*
1916	Yule	3rd ed. *An Introduction to the Theory of Statistics*
1917	Rugg	*Statistical Methods Applied to Education (abbrev.)*
1917	Secrist	1st ed. *Introduction to Statistical Methods*
1917	Yule	4th ed. *An Introduction to the Theory of Statistics*
1919	Yule	5th ed. *An Introduction to the Theory of Statistics*
1920	Bowley	4th ed. *Elements of Statistics*
1920	Bowley	3rd ed. *An Elementary Manual of Statistics*
1920	Elderton	3rd ed. *Primer of Statistics*
1920	Secrist	*Readings and Problems in Statistical Methods*
1920	Secrist	*Statistics in Business: their analysis, charting and use*
1921	Czuber	*Die Statistischen Forschungsmethoden*
1921	Jones	1st ed. *A First Course in Statistics*
1922	Davies	*Introduction to Economic Statistics*
1922	Yule	6th ed. *An Introduction to the Theory of Statistics*
1923	Elderton	4th ed. *Primer of Statistics*
1923	Kelley	*Statistical Method*
1924	Jerome	*Statistical Method*
1924	Jones	2nd ed. *A First Course in Statistics*
1924	Kent	*Elements of Statistics*
1924	King	2nd ed. *The Elements of Statistical Methods*

1924	Mills	1st ed. *Statistical Methods Applied to Economics and Business*
1924	Riegel	*Elements of Business Statistics*
1924	Yule	7th ed. *An Introduction to the Theory of Statistics*
1925	Chaddock	*Principles and Methods of Statistics*
1925	Crum	*An Introduction to the Methods of Economic Statistics*
192511	Fisher	1st ed. *Statistical Methods for Research Workers*
925	Gavett	1st ed. *A First Course in Statistical Method*
1925	Niceforo	*La Methode Statistique et ses Applications aux sciences naturelles (abbrev.)*
1925	Otis	*Statistical Method in Educational Measurements*
1925	Secrist	Rev. ed. *Introduction to Statistical Methods*
1925	Sutcliffe	*Elementary Statistical Methods*
1925	Thurstone	*Fundamentals of Statistics*
1926	Bowley	5th ed. *Elements of Statistics*
1926	Garrett	*Statistics in Psychology and Education*
1927	Elderton	2nd ed. *Frequency Curves and Correlation*
1927	Elderton	5th ed. *Primer of Statistics*
1927	Van Zanten	1st ed. *Leerboek der Statistische Methode*
1927	Yule	8th ed. *An Introduction to the Theory of Statistics*
1928	Bowley	4th ed. *An Elementary Manual of Statistics*
1928	Fisher	2nd ed. *Statistical Methods for Research Workers*
1928	Holzinger	*Statistical Methods for Students in Education*
1928	Verrijn Stuart	2nd ed. *Inleiding tot de beoefening der statistiek*
1929	Banister	*Elementary Applications of Statistical Method*
1929	Florence	*The Statistical Method in Economics and Political Science*
1929	Jones	2nd ed. rev. *A First Course in Statistics*
1929	Lindquist	*Study Manual in Elementary Statistics*
1929	Yule	9th ed. *An Introduction to the Theory of Statistics*
1930	Fisher	3rd ed. *Statistical Methods for Research Workers*
1930	Mills	2nd ed. *Statistical Methods Applied to Economics and Business*
1932	Fisher	4th ed. *Statistical Methods for Research Workers*
1932	Yule	10th ed. *An Introduction to the Theory of Statistics*
1933	Davies	*Methods of Statistical Analysis in the Social Sciences*
1933	Rhodes	*Elementary Statistical Methods*
1934	Arkin	*An Outline of Statistical Methods as applied to Economics, Business (abbrev)*
1934	Bowley	5th ed. *An Elementary Manual of Statistics*
1934	Fisher	5th ed. *Statistical Methods for Research Workers*
1934	Richardson	*An Introduction to Statistical Analysis*
1935	Bayliss	*A Course in Business Statistics: the elements of statistical methods (abbrev)*
1935	Fisher	1st ed. *The Design of Experiments*
1935	Kramer	*A First Course in Educational Statistics*
1935	Odell	*Statistical Method in Education*
1936	Davenport	4th ed. *Statistical Methods in Biology, Medicine, and Psychology*
1936	Elderton	5th ed. *Primer of Statistics*
1936	Fisher	6th ed. *Statistical Methods for Research Workers*
1936	Secrist	Rev. ed. *Introduction to Statistical Methods*
1936	Wheldon	*Business Statistics and Statistical Method*
1937	Bowley	6th ed. *Elements of Statistics*
1937	Fisher	2nd ed. *The Design of Experiments*
1937	Garrett	2nd ed. *Statistics in Psychology and Education*
1937	Gavett	2nd ed. *A First Course in Statistical Method*
1937	Reebs	*The Elements of Statistical Method for Students in Elementary Education*
1937	Yule	11th ed. *An Introduction to the Theory of Statistics*

(The Table excludes some authors mentioned by Bowley whose books we have been unable to trace.)

TABLE S2:Content of the three editions of Pearl's *Introduction to Medical Biometry and Statistics*

Chapter	Title	Start page		
		1st	2nd	3rd
I	Preliminary Definitions and Orientation	17	17	1
II	Some Landmarks in the History of Vital Statistics (Biostatistics)	27	42	21
III	The Raw Data of Biostatistics	45	63	42
IV	Tabular Presentation of Statistical Data	74	107	70
V	Origin of Scientific Records and Their Translation to Tabular Form	89	121	82
VI	Graphic Representation of Statistical Data	105	164	126
VII	Rates and Ratios	145	204	173
VIII	Life Tables	177	238	219
IX	Standardized and Corrected (Adjusted) Death Rates	198	263	269
X	The Probable (Sampling) Error Concept	209	278	282
XI	Elementary Theory of Probability	220	288	299
XII	Some Special Theorems in Probability	247	315	322
XIII	The Measurement of Variation	264	335	340
(XIV)	The Measurement of Variation (continued)	-	-	376
XIV (XV)	The Measurement of Correlation	292	366	409
XV (XVI)	Paired Correlation	319	394	437
XVI (XVII)	Simple Curve Fitting	332	407	448
XVII (XVIII)	The Logistic Curve	-	417	459
App. I	Aids to the Biometrics Worker		429	471
App. II	(Mathematical constants)		429	471
App. III	(Tables for Estimating the Significance of Deviations)		438	472
App. IV	(Table of Areas and Ordinates of the Normal Curve)		440	474
App. V	(Sum of Logarithms)		446	489
App. VI	(Reed-Merrell Tables for Abridged Life Table Construction)		-	492
App. VII	(Tables for Probable Errors of Differences between Means)		-	505
App. VIII	(Correction factors for the standard deviation and probable errors of mean and standard deviations of small samples)		-	512
App. IX	(A table for ascertaining elapsed time in years and decimals of a year between any two dates)		-	513
App. X	(A table of √PQ for computing the errors of simple sampling)		-	517
-	*Index*		449	521
-	*End*		459	537

(Items in brackets are changes from the second to the third editions)

TABLE S3

Hill *Principles of Medical Statistics* (Postgraduate Series Volume Three)

Edition	Year of Publication	Reprinted	Chapters
Lancet papers	1937	-	
1st	1937	-	17
2nd	1939	-	18
3rd	1942	1945,	18
4th	1948	1946	20

5th	1950	1949	
6th	1955	1952	
7th	1961	1959	
8th	1966	1962	
9th	1971	1967	
10th *	1977	1974	25
11th *	1985	-	
12th **	1991	-	

* *A Short Textbook of Medical Statistics*
** *Hill's Principles of Medical Statistics*, with I. D. Hill.

Table S4
Hill's 1937 Lancet Articles, 'Principles of Medical Statistics'

**I**	The aim of the statistical method	***Lancet* 1**, 41-43
**II**	Selection	***Lancet* 1**, 99-101
**III**	Presentation of results	***Lancet* 1**, 161-163
**IV**	The variability of observations	***Lancet* 1**, 219-221
**V**	Problems of sampling: averages	***Lancet* 1**, 281-284
**VI**	Further Problems of sampling: proportions	***Lancet* 1**, 337-340
**VII**	Further problems of sampling: differences	***Lancet* 1**, 402-405
**VIII**	Further problems of sampling: χ^2	***Lancet* 1**, 459-461
**IX**	Further examples and discussion of χ^2	***Lancet* 1**, 527-529
**X**	The coefficient of correlation	***Lancet* 1**, 583-586
**XI**	Life tables and survival after treatment	***Lancet* 1**, 646-648
**XII**	Common fallacies and difficulties	***Lancet* 1**, 706-708
**XIII**	Further fallacies and difficulties	***Lancet* 1**, 771-773
**XIV**	Further fallacies and difficulties	***Lancet* 1**, 825-827
**XV**	General summary and conclusions	***Lancet* 1**, 883-885
**XVI**	Calculation of the standard deviation	***Lancet* 1**, 941-943
**XVII**	Calculation of the correlation coefficient	***Lancet* 1**, 1001-1003

APPENDIX A

Publications of Hilda Mary Woods and William Thomas Russell

Hilda Mary Woods

Nine publications (in order of presentation) submitted for her Doctorate in Science in May 1933

Woods HM. Epidemiological Study of Scarlet Fever in England and Wales since 1900. *MRC Special Report Series* No. 180, HMSO, 1933. [acknowledges both Greenwood and Russell].

Woods HM. On the statistical epidemiology of respiratory disease. *Lancet* 1928: 17 March:539-? [Woods is assistant lecturer]

Woods HM. Statistical study of scarlet fever and diphtheria. *Journal of Hygiene* 1928:XXVIII(2):147-162. [acknowledges Greenwood and McKinlay]

Woods HM. The influence of external factors on the mortality from pneumonia in childhood and later adult life. *Journal of Hygiene* 1927:XXVI(1):37-43. [significance of correlation coefficients]

Woods HM. A note on the graphic method of graduation in the construction of life-tables. *Lancet* 1929:4 May:941-942. [Woods is assistant lecturer].

Greenwood M, Woods HM. "Status thymico-lymphaticus" considered in the light of recent work on the thymus. *Journal of Hygiene* 1927:XXVI(3):305-326.

Durham FM, Woods HM. Alcohol and inheritance: an experimental study. Medical research Council Special Report Series No. 168, 1932.

Woods HM, Stallybrass. The part played by meterological conditions on respiratory mortality in Liverpool. *Journal of Hygiene* 1932:XXXII(1):67-78.

Greenwood R, Thompson CM, Woods HM. Heights and weights of patients in mental hospitals. *Biometrika* 1925:XVII(I+2):142-158. [Rosa Greenwood is presumably Major Greenwood's wife]

Other papers not included in her thesis

Fowke H. The effect of supplements of vitamins and minerals on the health of girls. *British Medical Journal* 1943: 23 Oct:519.

Fowke HM. Discussion of *The statistical validity of methods used in budgetary and dietary surveys* by Greenwood M (*Proceedings of the Nutrition Society* 1945:3:23-28), *Proceedings of the Nutrition Society* 1945:3:28-30.

William Thomas Russell

Twenty-five publications (in order of presentation) submitted for his Doctorate in Science in 1943

Russell WT. The Epidemiology of Diphtheria during the last forty years. Medical Research Council *Special Report Series* No 247, HMSO, 1943.

Russell WT. The influence of fog on mortality from respiratory diseases. *Lancet*, 16 August 1924, 335.

Russell WT. The relative influence of fog and low temperature on the mortality from respiratory disease. *Lancet*, 27 Nov 1926, 1128-1130.

Young M, Russell WT. Some observations on the recorded mortality from diabetes in recent years in England and Wales as a whole and principal divisions, including London, with special reference to the introduction of insulin. *Quarterly Journal of* Medicine 1926:20:

Young M, Russell WT. A study of the longevity of males at different periods in the history of Great Britain and Ireland from the sixteenth century, based on data from the "Dictionary of National Biography" and "Burke's Peerage and Baronetage". *Journal of Hygiene* 1926:XXV (3):

Young M, Russell WT. Sexual differentiation in susceptibility to and mortality from whooping-cough in children under five years. *British Journal of Children's Diseases* 1927:

Young M, Russell WT. An investigation into the statistics of cancer in different trades and professions. Medical Research Council *Special Report Series*, No. 99, HMSO, 1926.

Russell WT. A study of Irish fertility between 1870 and 1911. *Metron* 1928:VII(2).

Russell WT. Dependency, orphanhood and fertility in England and Wales in 1921. *Sonderabdruck aus: Archiv für Soziale Hygiene und Demographie* 1929.

Goodall EW, Greenwood M, Russell WT. Scarlet fever, diphtheria and enteric fever 1895-1914: a clinical statistical study. Medical Research Council *Special Report Series*, No. 137, HMSO, 1929.

Dunkin GW, Hartley P, Lewis-Faning E, Russell WT. A comparative biometric study of albino and coloured guinea-pigs from the point of view of their suitability for experimental use. *Journal of Hygiene* 1930:XXX (3).

Russell WT. A review of the cancer statistics in England and Wales and in Scotland between 1891 and 1927. *Journal of Hygiene* 1931:XXXI (3).

Russell WT. Mortality from cancer according to site in the counties of Scotland 1923-8. *Journal of Hygiene* 1931:XXXI (4).

Russell WT. The statistics of erysipelas in England and Wales. *Journal of Hygiene* 1933:XXXIII (3).

Russell WT, Salmon G. Pulmonary tuberculosis in Wales between 1911 and 1931. *Journal of Hygiene* 1934:XXXIV (3).

Russell WT. Statistical analysis of the results of adult serum in the prevention and attenuation of measles. London County Council Measles Report, July 1933, page 77.

Russell WT. Statistical study of the sex ratio at birth. *Journal of Hygiene* 1936:XXXVI (3):381-401.

Gunn W, Russell WT. Immune measles sera in the control of measles outbreaks in the Council's hospitals, institutions and residential schools, during the measles epidemic 1st November 1933 to 31st August 1934. London County Council Measles Report, January 1936, page 24.

Greenwood M, Russell WT. Bright's disease, nephritis and arterio-sclerosis: a contribution to the history of medical statistics. *Biometrika* 1938:XXIX (III and IV).

Gunn W, Russell WT. A review of the measles epidemic 1935-3? Including reference to treatment and the preparation and use of immune measles sera. London County Council Measles Report 1938, page 19.

Cheeseman EA, Martin WJ, Russell WT. Disease and environment. *Biometrika* 1939:XXX (III and IV):341-362.

Cheeseman EA, Martin WJ, Russell WT. Diphtheria: a suggested explanation of the relative change in age incidence. *Journal of Hygiene* 1939:XXXIX (2):181-202.

Young M, Russell WT. Appendicitis. Medical Research Council *Special Report Series*, No. 233, 1939.

Greenwood M, Martin WJ, Russell WT. Deaths by violence 1837-1937 (with Discussion). *Journal of the Royal Statistical Society* 1941:CIV(II):146-171.

Aslett EA, D'Arcy Hart P, Martin WJ, Russell WT. Physical type in pneumoconiosis. *Journal of Hygiene* 1941:XLI(2).

Other publications not included in his thesis

Russell WT. The anthropometry of the population of a London borough (Hornsey). *Journal of the Royal Anthropological Institute* 1942:LXXII:19-22. [Has Russell as DSc]

Russell WT, WJ Martin. Dietetics of pregnancy. *British Medical Journal* 1943:1:204 and 1943:1:301 (letters).

Russell WT, Whitwell GPB, Ryle JA. Studies in occupational morbidity (I). *British Journal of Industrial Medicine* 1947:4:56-61.

Ryle LA, Russell WT. Social and occupational factors in the aetiology of skin cancer. *British Medical Journal* 1947:1:873-877.

Ryle JA, Russell WT. The natural history of coronary disease: a clinical and epidemiological study.*British Heart Journal* 1949:11:370-389.

Russell WT, Sutherland I. Mortality amongst babies from injuries at birth. *British Journal of Social Medicine* 1949:3:85-94.

Moloney GE, Russell WT, Wilson DC. Appendicitis: a report on its social pathology and recent surgical experience. *British Journal of Surgery* 1950:38:52-64.
